# Design, validation and implementation of an automated e-alert for acute kidney injury: 6-month pilot study shows increased awareness

**DOI:** 10.1186/s12882-023-03265-4

**Published:** 2023-07-27

**Authors:** Michael S.A. Niemantsverdriet, Wouter M. Tiel Groenestege, M. Khairoun, Imo E. Hoefer, Wouter W. van Solinge, Domenico Bellomo, Martin H. van Vliet, Karin A.H. Kaasjager, Saskia Haitjema

**Affiliations:** 1 Central Diagnostic Laboratory, University Medical Center Utrecht, Utrecht University, Room number G03.551, UMC Utrecht, Heidelberglaan 100, Utrecht, 3584 CX The Netherlands; 2 Lichtenauerlaan 40 3062ME, SkylineDx, Rotterdam, The Netherlands; 3 Department of Nephrology and Hypertension, University Medical Center Utrecht, Utrecht University, Heidelberglaan 100, Utrecht, 3584 CX The Netherlands; 4 Department of Internal Medicine and Acute Medicine, University Medical Center Utrecht, Utrecht University, Heidelberglaan 100, Utrecht, 3584 CX The Netherlands

**Keywords:** AKI, Electronic health records, E-alert, Before-after study, Data science

## Abstract

**Background:**

Acute kidney injury (AKI) is defined as a sudden episode of kidney failure but is known to be under-recognized by healthcare professionals. The Kidney Disease Improving Global Outcome (KDIGO) guidelines have formulated criteria to facilitate AKI diagnosis by comparing changes in plasma creatinine measurements (PCr). To improve AKI awareness, we implemented these criteria as an electronic alert (e-alert), in our electronic health record (EHR) system.

**Methods:**

For every new PCr measurement measured in the University Medical Center Utrecht that triggered the e-alert, we provided the physician with actionable insights in the form of a memo, to improve or stabilize kidney function. Since e-alerts qualify for software as a medical device (SaMD), we designed, implemented and validated the e-alert according to the European Union In Vitro Diagnostic Regulation (IVDR).

**Results:**

We evaluated the impact of the e-alert using pilot data six months before and after implementation. 2,053 e-alerts of 866 patients were triggered in the before implementation, and 1,970 e-alerts of 853 patients were triggered after implementation. We found improvements in AKI awareness as measured by (1) 2 days PCr follow up (56.6–65.8%, p-value: 0.003), and (2) stop of nephrotoxic medication within 7 days of the e-alert (59.2–63.2%, p-value: 0.002).

**Conclusion:**

Here, we describe the design and implementation of the e-alert in line with the IVDR, leveraging a multi-disciplinary team consisting of physicians, clinical chemists, data managers and data scientists, and share our firsts results that indicate an improved awareness among treating physicians.

**Supplementary Information:**

The online version contains supplementary material available at 10.1186/s12882-023-03265-4.

## Introduction

Acute kidney injury (AKI) is a sudden drop in kidney function that can develop within a few hours to days [1]. AKI incidence has increased over the past years and affects more than 20% of hospitalized patient; it, is associated with an increased risk of chronic kidney disease, longer hospital stay and mortality [2]. Despite the increased morbidity and mortality, AKI is often under-recognized and under-documented. Therefore, the ability to detect AKI episodes can improve outcomes and management of these patients, since there is no treatment available apart from reducing the causes (e.g. heart failure or infection) and triggers (e.g. administration of nephrotoxic medication) [3].

To facilitate (early) diagnosis, multiple guidelines, such as the Kidney Disease Improving Global Outcomes (KDIGO), have been proposed to provide diagnostic criteria that evaluate changes in plasma creatinine (PCr) [4], [5]. However, the KDIGO criteria are not routinely applied by physicians, hence AKI is often unrecognized. Without awareness and subsequent intervention(s), AKI can progress into chronic kidney disease (CKD) and require renal replacement therapy, thereby negatively influencing patients’ outcomes, costs and quality of live.

To improve AKI awareness, the KDIGO criteria have been implemented before as a rule-based algorithm in electronic health record systems (EHR) as an electronic alert (e-alert) for automated AKI diagnosis. Most algorithms use changes in PCr to alert the treating physician via various communication systems (e.g. text message, email, message in the EHR system or phone). Several randomized controlled trials (RCT), and before-after studies have been conducted to study the clinical value of these e-alerts, though with varying results. Haase et al. (2017) reviewed 15 studies, both non-RCT and RCTs, and found improvements in care processes (e.g. stop of adjustment in nephrotoxic medication) and lower mortality in some studies [6]. The heterogenous study designs as well as the various settings, may be the reason for these conflicting results.

Another reason may be the shallow design of the e-alert. Haase et al. showed that not all e-alerts provide concrete treatment recommendations or interrupt the treating physician’s routine clinical practice to draw attention to the alert. Even though a consensus paper of the Acute Dialysis Quality Initiative highlighted the use of e-alerts with specific treatment recommendations [7]. Moreover, it is unclear how studies designed their e-alert and whether this was done in close collaboration with the end-users as this may improve the success of an e-alert.

In a multidisciplinary team of clinical chemists, medical data scientists, data managers and physicians, we designed an e-alert for automated AKI diagnosis and implemented the e-alert in our EHR system at the University Medical Center Utrecht (UMCU), a tertiary hospital in The Netherlands. By close collaboration within a team of professionals, we aimed for three objectives: (1) keep the e-alert’s burden as low as possible for both stakeholder and end-users; (2) create clinical value for patients, and (3) develop and validate the e-alert in line with the IVDR. We performed a 6-monts before-after study using routine care data to assess both the burden, as well as the awareness of treating physicians by studying their actions.

## Methods

### AKI diagnosis flowchart

We designed a rule-based algorithm for automated AKI diagnosis that evaluates the three criteria, regarding PCr, adapted from the KDIGO guidelines (Supplemental Materials Fig. 1). These criteria assess changes in PCr as a difference (delta) between a PCr measurement and a previous ‘baseline’ PCr. AKI is diagnosed if there is an increase in PCr of 26,5 µmol/L within 48h, or an increase of 1.5 times ‘baseline’, which is known or presumed to have occurred in the previous seven days. Any value in the previous seven days is defined as ‘baseline’ and used to compute the ratio with a newly measured PCr. If more than one PCr measurement in the past 7 days was available, the lowest value was used as baseline to determine the delta PCr. If a patient did not have a PCr measurement available in the last 7 days, the algorithm selected the most recent PCr before the seven days to a maximum of one year as baseline [8]. The other two AKI stages (AKI stages II and III) were not further defined by the algorithm.

**Fig. 1:**
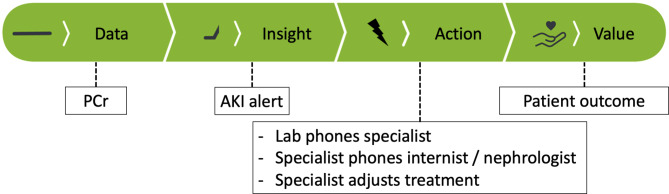
The four different stages of the AKI e-alert signalling cascade. PCr measurements are continuously generated and assessed by the AKI alert flowchart what may result into an actionable insight. This insight creates awareness that is turned into an action by the suggestions in the memo, after the physician has been alerted via phone and the EHR system. Depending on action taken, the alert may generate value for the patient

### AKI e-alert

For every new PCr measurement, the flowchart was evaluated by our Laboratory Information System (LIS) (GLIMS 9.9.6, Clinisys, Gent, Belgium). When the e-alert was triggered, the physician who requested the PCr was notified via two ways: (1) the e-alert outcome was sent to and presented in our EHR as an additional ‘measurement’ in the laboratory test results page, and (2) a laboratory technician phoned the physician to notify that the requested PCr measurement triggered the e-alert. The e-alert outcome shown in the EHR (HiX 6.1, Chipsoft B.V., The Netherlands, Amsterdam) for the AKI ‘measurement’ was either “positive” or “unable to compute”. No outcome was shown when the alert’s outcome was negative (no AKI). To make the e-alert as actionable as possible, a text box (memo) was shown when the physician hovered with the mouse cursor over the outcome in the EHR system (Box 1, Box 2) as shown in Supplementary Materials Figs. 2 and 3. The text was mainly adapted from the KDIGO guidelines and aligned with our in-house work philosophy [9]. In addition, the memo referred to the hospital-wide AKI protocol in our hospital’s protocol system ‘iProva’ where both AKI in general and the e-alert system are explained, as well the definition of nephrotoxic medication adapted from Ashley et al. (2015) as listed in Supplemental Materials Table 1 [10].

**Table 1:** AKI episode characteristics in the before and after periods. *: AKI episodes filtered based on use of nephrotoxic medication as not all patients with AKI were using nephrotoxic medication. Only p-values are shown for outcomes related to awareness

	Before (6th of April 2021 – 5th of October 2021)	After (6th of October 2021–5th of April 2022)	p-value
Episodes	884	819	
Department at start episode - Emergency department - Hospital ward - Outpatient department	147 (13.6%) 492 (45.6%) 245 (22.7%)	184 (18.1%) 463 (45.6%) 172 (16.9%)	
PCr follow up within 2 days - Emergency department - Hospital ward - Outpatient department	500/884 (56.6%) 103/147 (70.1%) 344/492 (69.9%) 53/245 (21.6%)	538/819 (65.7%) 155/184 (84.2%) 346/463 (74.7%) 37/172 (21.5%)	< 0.001 0.003 0.11 1.0
Episodes with nephrotoxic medication count(%)			
- Emergency department - Hospital ward - Outpatient department	83/147 (56.5%) 341/492 (69.3%) 99/245 (40.4%)	107/184 (58.2%) 340/463 (73.4%) 71/172 (41.3%)	
Stop medication within 7 days* - Emergency department - Hospital ward - Outpatient department	407/523 (77.8%) 76/83 (91.6%) 288/341 (84.5%) 43/99 (43.4%)	442/518 (85.3%) 100/107 (93.5%) 297/340 (87.4%) 45/71 (63.4%)	0.002 0.83 0.33 0.016

When a patient’s PCr measurements generated multiple e-alerts within one week, only the first AKI episode would be communicated by phone, yet all would be displayed in the EHR. If the patient developed an AKI episode one week after the first alert, a laboratory technician would contact the treating physician again. When a patient met the AKI criteria on day 1 and on day 3, with a PCr measurement on day 2 that did not trigger the e-alert, a laboratory technician would phone the physician both on day 1 and day 3. The e-alert was shown in the EHR system for patients older than 18 years that were treated by all departments, except for the dialysis department (as per request). In addition, all departments except for the dialysis, ICU or COVID-19 wards were phoned (as per request by these departments), as patients were already under high medical surveillance in these wards.

### Co-creation with stakeholders

After we developed the e-alert system, we pitched our idea to the laboratory, internal medicine and nephrology departments to receive feedback, as well as to ask how they wanted to be part of the alerting system. We further collaborated with the specialty departments who would most likely be phoned for consult after the onset of an e-alert, as we mentioned them in the memo text: nephrology, internal medicine and the pharmacy. From these stakeholder sessions, we gathered input to finalize the design phase before starting the implementation phase.

### Implementation and validation phases

As the result of the e-alert was part of diagnostic decision making, the e-alert classifies as a *class B Software as a Medical Device* (SaMD) according to the IVDR [11]. According to the regulation, developed in-house tests have to meet both safety and performance requirements, and specifically software has to meet development and manufacturing requirements. Our lab is compliant with the ISO 15189 quality management standard to ensure safety and performance. We further verified and validated our software in line with the IEC 62304 standard [12].

To validate the functioning of the flowchart, we performed a risk analysis where we applied the flowchart on various PCr measurements to analyse the risks in terms of false positives and false negatives, and adjusted the system accordingly. Subsequently, we verified the e-alert by evaluating whether the implemented code generated the expected results by creating ‘shadow’ alerts in the background of our LIS system. The complete system was tested by extracting all data of the shadow alerts from the LIS together with all PCr measurements of patients that had at least one e-alert during the validation phase.

### Before-after study to assess increase in AKI awareness

We performed a 6-month before-after study to study the effect in awareness of the e-alert in our hospital. We defined two patient groups by selecting all patients six months before (before group 6th of April 2021 to 5th of October 2021) and six months after the implementation date of the e-alert (after group, 6th of October 2021 to 5th of April 2022). At the end of 2021, patients who required intensive treatment were dedicated to the COVID cohort. These patients were excluded from the analysis (N = 55) as they received different treatment as compared to other departments. From 2022 onwards, patients with COVID were admitted to “normal” wards, and thereby automatically included in our analysis.

As the final version of the e-alert was already implemented in the background of the LIS during the testing phase, we could easily identify the patients who triggered an e-alert both before the alert was introduced (shadow alerts), as well as after (real alerts). For these patients, we extracted demographic data, all PCr measurements and all administered nephrotoxic medication from the Utrecht Patient Oriented Database (UPOD) to assess if actions were taken by treating physicians. In brief, UPOD is an infrastructure of relational databases comprising data on patient characteristics, hospital discharge diagnoses, medical procedures, medication orders and laboratory tests for all patients treated at the UMCU since 2004 [13]. PCr was measured by an enzymatic isotope dilution mass spectrometry traceable assay on an Atellica CH analyzer (Siemens Healthcare Diagnostics Inc., Tarrytown, USA).

### AKI episode and endpoints

Treating physicians may receive multiple consecutive e-alerts for the same patient. Therefore, performing analyses on all e-alerts would not be a valid representation of the system as the physician is not phoned when a second alert is generated within one week, and may already have changed treatment after receiving the first alert. For this reason, we focused on the first e-alert and defined this as the start of an AKI episode. To investigate whether the introduction of the e-alert led to action and awareness, we used follow up measurements of PCr within 2 days and discontinuation of at least one nephrotoxic medication within 7 days after the start of AKI episode. Patients who did not use any nephrotoxic medication were excluded from the second analysis. We also assessed the awareness on different levels by stratifying all analyses according to sex of the treated patient, hospital department (emergency department, hospital ward and outpatient clinic) and specialty of the treating physician. All data pre-processing and analyses were performed using the R environment (version 4.2.0). p-values were computed with the Chi-Square test, to test between the proportion of AKI episodes that were followed by an action, between the before and after period.

## Results

### Feedback from co-creation sessions

After the co-creation sessions, we assessed the feedback and made amendments accordingly to our AKI alert signaling system. For example, together with the intensive care unit (ICU), we decided to leave out the ICU department from being phoned by the laboratory, as ICU patients are already under high surveillance. As laboratory technicians were concerned about the potential high number of phone calls they had to make, we co-decided to add the one-week phone call delay between subsequent AKI alerts during the same episode to reduce the number of phone calls from the laboratory. These changes led to the final version of our alert system as described in the Methods section above.

### Before and after periods show similar patient and alert characteristics

On October 6, 2021, we implemented the e-alert in our EHR system. For both the before and after periods, we found a similar number of patients for whom the e-alert triggered at least once (866 and 853 respectively), and a comparable number of alerts (2,053 and 1,970 respectively). Comparing the two periods, patients were of similar age on the day of the first alert (60.3±16.3 vs. 60.0±16.8), and the percentage of women was also similar (46.8% vs. 45.7%) (Supplemental Materials Table 2). Sixty-three patients were included in both periods. We found similar patient characteristics of the AKI episodes in terms of clinical department of the treating physician. After removing the AKI episodes of patients admitted to the ICU or COVID-19 cohort, 884 AKI episodes in the before period, and 819 episodes in the after period remained. Of all 819 episodes in the after period, only once the laboratory technicians did not phone the physician. The turnaround time of PCr in the laboratory was under 60 minutes. Time between PCr measurements and subsequent phone call was on average within an hour (median of 12 minutes).

### Improved awareness in the after period

**Fig. 2:**
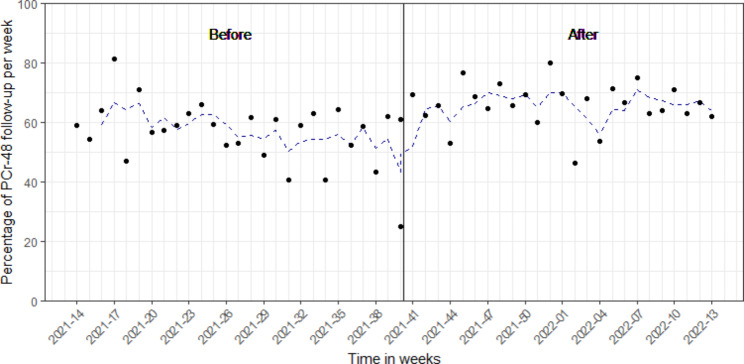
Percentage of AKI alerts per week that have been followed up with a PCr measurement within 48h between the before (6th of April 2021–5th of October 2021) and after period (6th of October 2021–5th of April 2022). The blue dashed line represents a 3-week moving average. The vertical line denotes the introduction of the AKI alert 6th of October, 2021

**Fig. 3:**
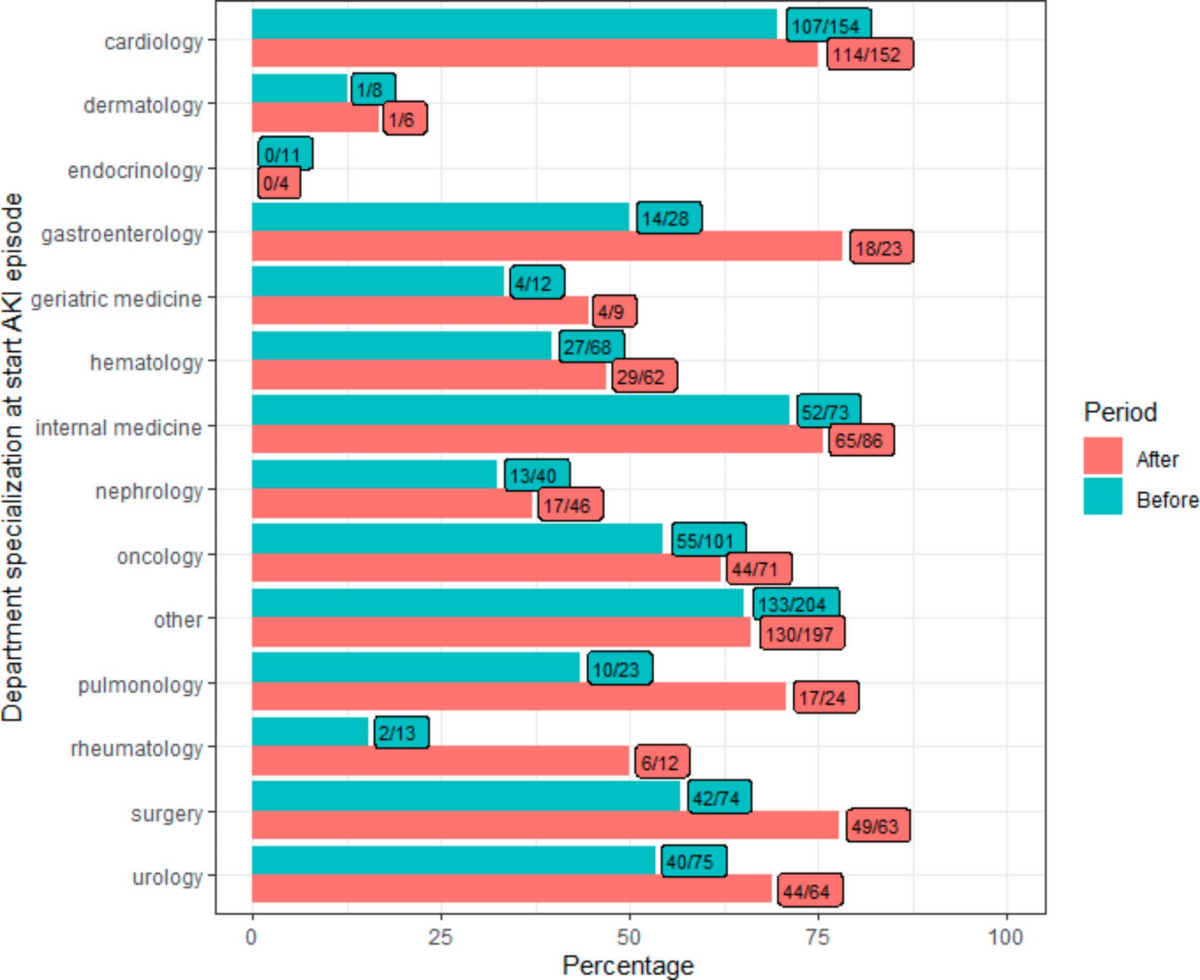
PCr follow up within 48h per treating specialty between the before (6th of April 2021–5th of October 2021) and after period (6th of October 2021–5th of April 2022). The horizontal bars visualise the percentage of AKI alerts that had a PCr follow up within 48h. Numbers in the boxes indicate how many times an alert had a PCr follow up, with respect to the total alerts per treating specialty (followed-up/total)

In total, the e-alert generated 16 phone calls from the treating physician to the internal medicine department in the after period to ask for treatment advice, the pharmacy was phoned once. We found a higher percentage of 48h PCr follow up in the after period as compared to the before period, 65.7% and 56.6%, respectively (p-value: <0.001) (Fig. 2; Table 1). When focusing on hospital departments, both the emergency department and hospital wards showed an increase in PCr follow up within 2 days between the before and after groups, from 70.1 to 84.2%, (p-value: 0.003) and from 69.9 to 74.7% (p-value: 0.110), respectively (Table 1). The percentage of follow up was similar for the outpatient clinics before and after implementation of the e-alert; 21.6% and 21.5%, respectively (p-value: 1.0) (Table 1). Overall, we observed an increase in PCr follow up when comparing treating specialties regardless of hospital department (emergency department, hospital wards and outpatient clinics) (Fig. 3). By stratifying for sex, we saw a small follow up increase in men (62.3 vs. 69.6, p-value: 0.024) and a larger increase in females (50.2% vs. 60.9%, p-value: 0.003).

**Fig. 4:**
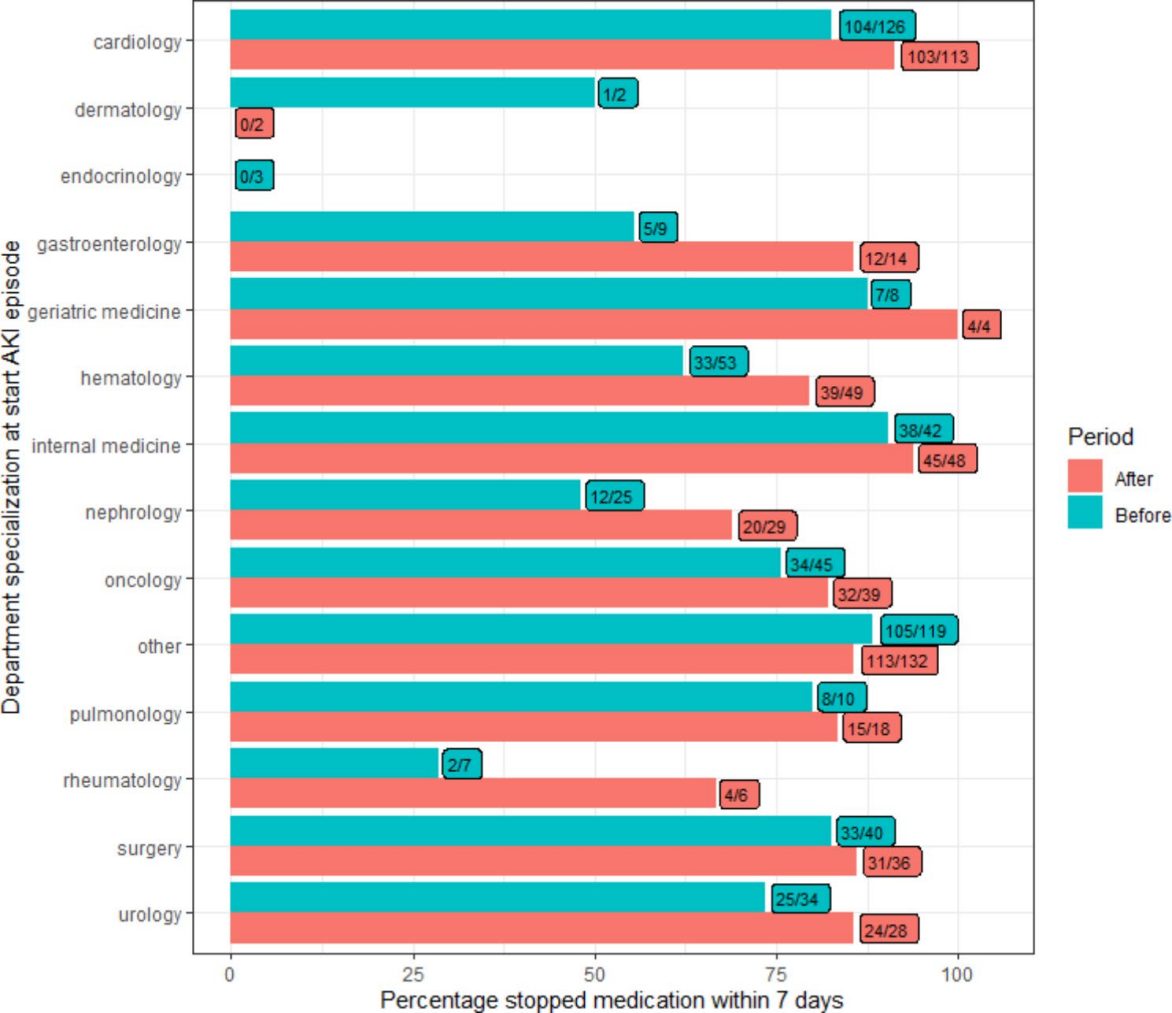
Nephrotoxic medication stopped within 7 days per treating specialty between the before (6th of April 2021–5th of October 2021) and after period (6th of October 2021–5th of April 2022). This figure only represents those patients who were using nephrotoxic medication at the start of the AKI episode. Numbers in the boxes indicate for how many alerts, at least one nephrotoxic medication was stopped per patient that had a PCr follow up, as compared to the total alerts per treating specialty **Box 1: memo text as shown in our EHR system when the AKI alert identified the patient as having AKI** *According to the KDIGO guidelines, this patient has acute kidney injury (AKI)* *Follow up the kidney function* *Be aware of the use of nephrotoxic medication and consider changing medication dosage. If necessary, please contact the pharmacist (phone number)* *For questions about kidney function deterioration, consider consult with the internal medicine (phone number) or nephrology department (phone number)* *For more info, please find the AKI-alert document in our iProva system* **Box 2: memo text as shown in our EHR system when the patient did not have a baseline PCr measurement** *Not able to evaluate according to the KDIGO guidelines due to no available PCr measurement in the previous 365 days*

Of all e-alerts in the before and after periods, 523 (59.2%) and 518 (63.2%), respectively, were related to patients who received nephrotoxic medication at the start of an AKI episode. We observed a trend towards nephrotoxic medication that was stopped within seven days throughout the hospital (77.8% vs. 85.3%, p-value: 0.002) and also within the three hospital departments; ED (91.6–93.5%, p-value: 0.83), hospital wards (84.5–87.4%, p-value: 0.33) and outpatient clinics (43.4–63.4%, p-value: 0.016). We did not observe a difference between specialty departments (Fig. 4). We found an increase for both males (81.4–85.8%, p-value: 0.186) as well as females (73.1–84.7%, p-value: 0.004), when comparing the number of stopped nephrotoxic medication within seven days between the before and after periods.

## Discussion

With a multi-disciplinary team, we developed an e-alert to detect AKI in patients who visit the UMCU. We designed an “actionable insight” consisting of a flowchart to automatically diagnose AKI according to KDIGO guidelines in our LIS, as well as an alert system with text in the EHR a phone call by our laboratory technicians and a link to the hospital wide AKI protocol. By co-creating with different stakeholders and end-users, we received valuable feedback with which we improved our alerting system to further foster AKI awareness. Furthermore, we designed, implemented and validated our system according to the new IVDR norms. The results show increased awareness and action of active intervention in clinical practice across the hospital in terms of 2 days PCr follow up and stop of nephrotoxic medication within 7 days after the start of the AKI episode.

To our knowledge, this is one of the few studies that describes a methodology of co-designing an e-alert for automated AKI diagnosis. Though there are several examples of other e-alerts that have been investigated, either in before-after studies or RCTs, only a small portion actually specifies the design and implementation of the e-alert [6, 14]. For example, the development of the National Health Service AKI alerting system had a similar, but nation-wide approach, by first discussing the need of an AKI e-alert during the UK Acute Kidney Injury Consensus Conference. This was followed by a rigorous exploration to scope the feasibility, by talking to various professional organizations and stakeholders, including LIS, laboratories and physicians [15]. This AKI alerting system is still in place, what may hint that collaboration leads to better foundation for the introduction, adoption and subsequent success of an alerting system.

When comparing e-alerts systems that are based on the KDIGO guidelines, two major elements stand out: the definition of baseline PCr and the type of alert. In line with our previous research, we defined baseline for patients without a baseline PCr measurement in the previous seven days, as the most recent value within 7 to 365 days [8]. This may have introduced false positives as an older PCr measurement may not be a good representation of the patient’s baseline. Even though, we found that the majority of e-alerts were based on baseline values from the previous seven days (3,189 of the 4,023 alerts, 79.3%). When compared to other e-alert designs, such as a pop up in the EHR or a clinical response team, we decided to phone the treating physician when the alert was triggered to stimulate the physician to inspect and adhere the KDIGO suggestions as shown in the EHR memo [16, 17]. As this is already part of our hospital’s system and work philosophy, where the laboratory communicates measurement results to the treating physician when exceeding predefined critical limits, our notifications were low-cost, practical to implement and kept the burden for both the laboratory and physicians to a minimum.

The development and validation of an e-alert in line with the IVDR regulation has not been widely shared in literature. As the IVDR has come into effect as of May 2022, every new in-house developed SaMD, such as our e-alert, should adhere to this regulation. We experienced that close collaboration within a multi-disciplinary team is key for the development and awareness of an e-alert [12]. As a result, every stakeholder was fully aware of the system’s functioning and its role within the system what reduced the risk for the patient. Moreover, by incorporating co-creation with stakeholders during the design phase, we were able to further shape our actionable insight according to feedback from other stakeholders. This augmented the functioning of the system and was useful for further adoption of the diagnostic support tool. By doing this, we continuously evaluated the ratio between the burden for both stakeholders and end-users, as well as the clinical value for patients.

After the launch of the alert, we observed 16 phone calls from the treating physician to the internal medicine department related to the alert over a six months’ time period. This number was only marginal compared to the total number of alerts in this period (N = 1970). On one hand, it showed awareness by physicians as suggested in the alert’s text. On the other hand, the nephrology and internal medicine departments did not experience additional burden after the alert’s implementation. We only registered the phone calls to the internal medicine and pharmacology departments, and not to the nephrology department. Overall, these departments did not experience additional burden related to the alert, although a follow up qualitative research on this aspect may provide more specific insights. However, we did not have a baseline of phone calls from the treating physician to the internal medicine department from the before period to make a good comparison in the number of phone calls related to AKI between both periods.

Though easily accessible and available, performing analyses on routine care data comes with many drawbacks that may weaken our findings [18]. As we did not have access to PCr values that were measured outside our hospital, we may have missed PCr follow up either before or after hospital visits, which may also be an explanation for the low numbers of follow up in the outpatient clinic. Likewise, we did not have information on medication use prescribed by other care providers which may also be an explanation for the low numbers of follow up in the outpatient clinic. Likewise, we did not have information on medication use prescribed by other care providers outside our hospital which may explain the differences between the in- vs. outpatient settings. Though, the medication use of patients admitted to our hospital and patients who visit the outpatient clinic is assessed by the medical professional.

Even though we observed some interesting changes in clinical care that are in line with the introduction of the e-alert, our before-after pilot study was only based on six months data. The before period was during summer and the after period during winter, which may have induced a seasonal effect. Moreover, in the winter of 2022 there was a steep increase in the number of COVID-19 patients in the province of Utrecht. Even though, the patients’ characteristics of the before and after period were alike and COVID-19 patients who were admitted to the COVID ward were excluded from the analysis (N = 55), the pandemic may have affected the standard practice in our hospital as COVID-19 in general disrupted our healthcare system.

As a follow up to this pilot study, we are interested in the experience of physicians working with the e-alert and may make additional changes to our design accordingly. Following the signalling cascade (Fig. 1), the next step would be to compare patients’ clinical outcomes, such as hospital length of stay and mortality, which warrants using a longer follow up period. Moreover, extending the range of available data may reduce both seasonal effects as well as the effect of COVID-19. As the KDIGO criteria do have limitations and the nephrology community is continuously extending their understanding of AKI and best practices, we may update our design accordingly when new guidelines are defined, while preserving IVDR compliancy [19, 20]. For example, additional diagnostic biomarkers for a more accurate diagnosis and AKI sub-phenotyping, when proven, might be added to the memo text as a suggestion to improve the determination of cause and prognosis [21].

Here, we shared both our experience as well as the steps for the design, implementation and validation phase of our e-alert in line with the IVDR regulation. We found that close collaboration within a multi-disciplinary team and further co-creation with a large group of stakeholders are major prerequisites for both the development as well as the implementation success of an e-alert. Though we share our blueprint for the development of a in house-made SaMD e-alert system, each e-alert should be tailored to the local site depending on work philosophy and protocols that are already in place followed by a validation and verification according to the local quality management systems and development protocols. Moreover, continuous internal evaluation is required to make changes to the system to keep the burden for care givers as low as possible while maximizing the clinical value for patients.

### Electronic supplementary material

Below is the link to the electronic supplementary material.

Supplementary Material 1

## Data Availability

The datasets generated and/or analysed during the current study are currently not publicly available due to patient privacy restrictions but are available from the corresponding author (s.haitjema@umcutrecht.nl) upon reasonable request.
