# Supplementary material for: Design, validation and implementation of an automated e-alert for acute kidney injury: 6-month pilot study shows increased awareness
Source: BMC Nephrol. 2023 Jul 27;24:222. doi: 10.1186/s12882-023-03265-4 (PMC10375640; doi:10.1186/s12882-023-03265-4)

**Supplemental Materials Table 1: medication defined as nephrotoxic medication adapted from Ashley et al. (2015) with Anatomical Therapeutic Chemical (ATC) codes.** *: all drugs with similar ATC codes were included.

| Type | Drug | ATC code |
| --- | --- | --- |
| antiviral | aciclovir | J05AB01 |
| antifungal | amphotericin B | J02AA01 |
| antibacterial | amikacin | J01GB06 |
| immunosuppressor | ciclosporin | L04AD01 |
| antiviral | foscarnet | J05AD01 |
| antiviral | ganciclovir | J05AB06 |
| antibacterial | gentamicin | J01GB03 |
| mood stabilizer | lithium | N05AN01 |
| immunosuppressor | methotrexate | L04AX03 |
| NSAID |  | MO1AB* |
| NSAID |  | MO1AC* |
| NSAID |  | MO1AE* |
| NSAID | acetylsalicylic acid | N02BA01 |
| immunosuppressor | sirolimus | L04AA10 |
| immunosuppressor | tacrolimus | L04AD02 |
| antibacterial | tobramycin | J01GB01 |
| antiviral | valaciclovir | J05AB11 |
| antiviral | valganciclovir | J05AB14 |
| antibacterial | vancomycin | J01XA01 |
| diuretics |  | C02L* |
| diuretics |  | C03* |
| ace inhibitors |  | C09* |
| angiotensin II receptor blocker |  | C09* |

**Supplemental Materials Table 2: patients characteristics of before and after periods.**

|  | **Before  (6^th^ of April 2021 – 5^th^ of October 2021)** | **After (6^th^ of October 2021 – 5^th^ of April 2022)** |
| --- | --- | --- |
| Patients, count | 866 | 853 |
| Age patient on the day of first alert, mean (SD) | 60.3 (16.3) | 60.0 (16.8) |
| Sex, female count (%) | 405 (46.8%) | 390 (45.7%) |
| Admitted at least once during period, count (%) | 692 (79.9%) | 722 (84.6%) |
| Alerts, count | 2,053 | 1,970 |
| Number of patients with *n* number of alerts, count   - 1 - 2 - >= 3 | 526 (60.7%)  128 (14.8%)  212 (24.5%) | 506 (59.3%)  133 (15.6%)  214 (25.1%) |


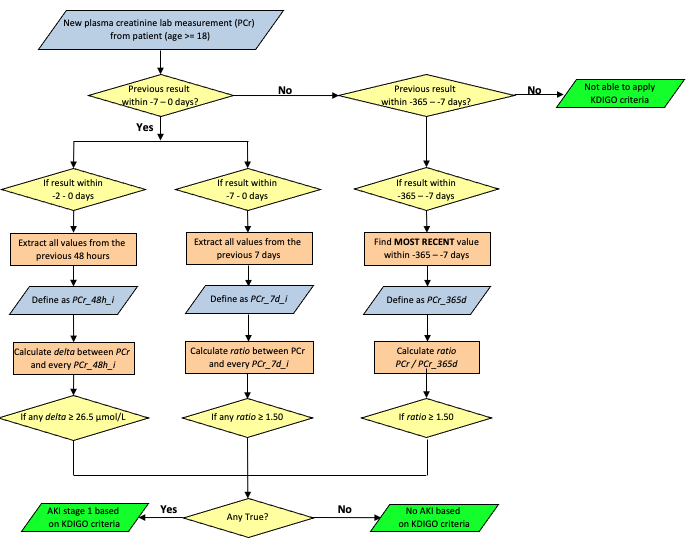


**Supplemental Materials Figure 1: AKI alert flowchart with the three diagnostic criteria adapted from the KDIGO guidelines.** For every new plasma creatinine (PCr) measurement from a patient older than 18 the flowchart is evaluated. Depending on the availability of a baseline measurement in the previous seven days the flowchart evaluates either two criteria or one criterion. If no PCr measurement is available in the previous 365 days, or when none of the criteria are met, the patient does not have AKI based on the KDIGO criteria.


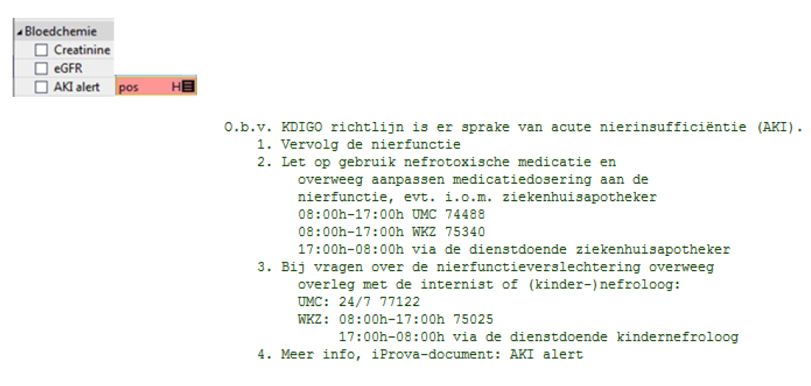
**Supplemental Materials Figure 2: visualization of our EHR system HiX where a patient was identified as having AKI**. When hovering with the mouse over the AKI alert result, the memo pops up, with the recommendations from the KDIGO guidelines in Dutch (English translation shown in Box 1).

**Supplemental Materials Figure 3: visualization of our EHR system HiX where a patient could not be evaluated by the AKI e-alert as not enough baseline plasma creatinine values were available to be used as baseline.** When hovering with the mouse over the AKI alert result, the memo pops up, with the explanation in Dutch that the AKI alert could not be computed due to no baseline plasmas creatinine measurements available (English translation shown in Box 2).


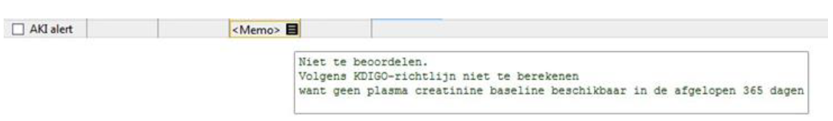

Supplement: Supplementary file 1 — Supplementary Material 1 [file 12882_2023_3265_MOESM1_ESM.docx]
